# Domestic Economy

**Published:** 1894-10-13

**Authors:** 


### The Institutional Workshop.

### HOSPITAL FINANCE.

### XI.—DOMESTIC ECONOMY.

Much has been done of recent years, both in this country and abroad, to check the waste commonly associated with the idea of large institutions and encourage a thrifty spirit in their domestic management. The introduction of the Uniform System of Accounts has effected more to this end in a short time than many years of incessant cavil and recrimination. The fact is that general slackness is responsible far more often than dishonesty for the flagrant instances of waste and extravagance which come to light from time to time in the expenditure of charitable monies. Easy-going amiability on the part of the persons chiefly responsible, which refrains from reproof or personal inspection as an impertinence towards subordinates; a bad method inherited from predecessors and never reformed, together with absolute ignorance of the importance of little things, will lose an institution in the course of years fully as much as a defaulting secretary can dare to purloin. And the mischief is perpetuated because no one is aware of what is wrong. The admirably simple system by which each hospital can now compare accounts with its neighbours makes it possible to see at once in which department the leakage exists, and to see, in the case of an honestly managed institution, is to set right. There is, perhaps, no branch of human affairs in which mutual comparison is more advantageous than in housekeeping, as shown by the endless discussion of such matters by the initiated. And this has become for the first time possible to the hospitals by the Uniform System of Accounts. The authorities at St. Mary's, for instance, can hardly fail to be puzzled that their provisions cost £32 a bed, as against £18 per bed at University. But when they compare accounts, and perceive that exactly three times the amount of eggs and butter (to mention no other items) is consumed per bed at St. Mary's than is consumed at University, and one-third more of the same articles than at Middlesex, even with its exceptional expenses, they are provided at once with a definite point for investigation.

There is one, and perhaps only one, point of accord, which strikes the student of the published rates of hospital provisioning. And that is the relatively lower cost of provincial, compared with metropolitan hospitals. Taking them all together, it will be found that the average per occupied bed is some £6 lower in the country than in London, while in Scotch and Irish hospitals the average is £2 lower again. This can hardly result from any other main cause than a difference in markets. The metropolitan hospitals evidently pay more for their goods than their provincial neighbours. That this need not be the case is amply proved by the experience of the numerous people living in the country or in country towns who find it cheaper to get their main food supplies from London. It would not, in fact, be easy to find any place where food can be had at a lower rate than in London, and it is natural to conclude that the metropolitan institutions labour under some grave disadvantage with regard to their system of food supplies, from which private persons are exempt. The system commonly in force is that of contract.* "In practice this system of contract works very unequally, and unless the authorities of an institution are fully alive to the state of the markets and to the necessity of instituting a system which will prevent the introduction of inferior articles and secure the immediate detection of any tampering with those who have it in their power to accept or reject the goods when delivered, experience shows that goods supplied by tender have a habit of steadily deteriorating." That large contracts do not necessarily ensure low prices is shown from the reports issued by the Local Government Board relative to the poor law infirmaries. "The prices show amazing differences. An item like potatoes, for instance, will cost 100 per cent. more at one institution than another. Rice, coffee, tea, and nearly every other item reveal the same differences. . . . We have further satisfied ourselves that price is very often no test of quality, and that sometimes an exorbitant price may be paid for a very indifferent article."

With all these drawbacks, as matters stand at present, the contract system is inevitable. The art of buying is not altogether so common an accomplishment as is generally supposed, and it is hardly too much to say that, unless the services of a professional buyer of first-rate ability were enlisted, the maximum of quality with the minimum of cost could never be attained. Moreover, the buyer must have it in his power to purchase at the right moment. But few institutions have space at their disposal which permits them to receive large consignments of goods. They are compelled to live in great measure from hand to mouth, and are therefore at the mercy of the middle man, who naturally secures the lion's share of the advantages derived from the size of the transactions.

These drawbacks are obviated in Paris by the agency of a central store for supplying the public institutions. The interests involved are even greater than would be the case in a corresponding organisation in London, since pauper asylums, almshouses, and public orphanages are included in its scope, in addition to hospitals for the sick, the whole embracing a population of over 500,000 persons, exclusive of staff. The admirable system of co-operation in force ensures not only a large economy, owing to the command of the market obtained, but also a high standard of quality and freedom from adulteration in articles requiring preparation before delivery. The following are some of the departments:—

(*a*) The central slaughter-house, founded in 1848, is a model institution which London would do well to imitate. A guarantee is required as to the condition of every animal received, and the arrangements, both as regards sanitation and humanity, are excellent. Might not some of our large institutions, as among the greatest consumers, take the lead in enforcing a much-needed reform in this direction?

(*b*) The central bakery in the Place Scipion turns out all the bread needed in the hospitals and almshouses

* "Hospitals of the World," by H. C. Burdett, page 181.

and by undertaking the actual grinding of the corn from the best crop renders adulteration impossible. The new system of exporting the corn from America ready ground, which has ruined so many English millers, would render this branch of the service unnecessary. As regards the after processes it is not at all clear that institutions entering upon large contracts of bread understand or accept their measure of responsibility for the conditions under which it is produced, and recent revelations have shown that investigation and continued supervision are nowhere more urgently called for than in bakeries.

(*c*) In 1864 a central dairy was organised in the outhouses of Bicêtre for the purpose of providing lying-in departments with good milk.

(*d*) The wine is bought at special contract by the central cellar, which prepares the blends with natural wines by the help of expert tasters.

(*e*) In 1851 a service was instituted to provide all commodities sold at the central markets, such as poultry, fish, butter, eggs, vegetables, &c. Each establishment applies for the quantity required, and as these commodities are bought wholesale or by public auction a great saving is effected, and a more varied diet is secured for the patients.

Several other large French towns have started a central depôt on a similar principle for the supply of the hospitals. In Brussels the Magasin Central obtains by contract the provisions necessary for the supply of the institutions, and undertakes the work of guaranteeing the quality of the goods, and distributing them as required. The hospitals are obliged to obtain all their supplies from this central store, which, as in Paris, undertakes the slaughtering, bread-making, &c., on its own premises.

The establishment of some such central service in London for the supply of hospitals and other institutions would doubtless work wonders in lowering the average cost of provisions, and, which is infinitely more important, in raising the standard of quality. It is far worse that patients should be ill-fed, than that the public should be called upon to pay a good deal more than is necessary to feed them. Both evils are the offspring of ignorance rather than of intention, and the existence of a department which should relieve the hospital authorities of the burden of commercial responsibilities would act with equally salutary force on the cheese-paring and the lavish administration.

